# Prevalence of Titin Truncating Variants in General Population

**DOI:** 10.1371/journal.pone.0145284

**Published:** 2015-12-23

**Authors:** Oyediran Akinrinade, Juha W. Koskenvuo, Tero-Pekka Alastalo

**Affiliations:** 1 Children’s Hospital Helsinki, Institute of Clinical Medicine, University of Helsinki and Helsinki University Central Hospital, Helsinki, Finland; 2 Blueprint Genetics, Helsinki, Finland; 3 Department of Clinical Physiology and Nuclear Medicine, HUS Medical Imaging Center, Helsinki University Central Hospital and University of Helsinki, Finland; University of Texas MD Anderson Cancer Center, UNITED STATES

## Abstract

**Background:**

Truncating titin (*TTN*) mutations, especially in A-band region, represent the most common cause of dilated cardiomyopathy (DCM). Clinical interpretation of these variants can be challenging, as these variants are also present in reference populations. We carried out systematic analyses of *TTN* truncating variants (TTNtv) in publicly available reference populations, including, for the first time, data from Exome Aggregation Consortium (ExAC). The goal was to establish more accurate estimate of prevalence of different TTNtv to allow better clinical interpretation of these findings.

**Methods and Results:**

Using data from 1000 Genomes Project, Exome Sequencing Project (ESP) and ExAC, we estimated the prevalence of TTNtv in the population. In the three population datasets, 52–54% of TTNtv were not affecting all *TTN* transcripts. The frequency of truncations affecting all transcripts in ExAC was 0.36% (0.32% - 0.41%, 95% CI) and 0.19% (0.16% - 0.23%, 95% CI) for those affecting the A-band. In the A-band region, the prevalences of frameshift, nonsense and essential splice site variants were 0.057%, 0.090%, and 0.047% respectively. *Cga/Tga* (arginine/nonsense–R/*) transitional change at CpG mutation hotspots was the most frequent type of *TTN* nonsense mutation accounting for 91.3% (21/23) of arginine residue nonsense mutation (R/*) at *TTN* A-band region. Non-essential splice-site variants had significantly lower proportion of private variants and higher proportion of low-frequency variants compared to essential splice-site variants (*P* = 0.01; *P* = 5.1 X 10^−4^, respectively).

**Conclusion:**

A-band TTNtv are more rare in the general population than previously reported. Based on this analysis, one in 500 carries a truncation in *TTN* A-band suggesting the penetrance of these potentially harmful variants is still poorly understood, and some of these variants do not manifest as autosomal dominant DCM. This calls for caution when interpreting TTNtv in individuals and families with no history of DCM. Considering the size of *TTN*, expertise in DNA library preparation, high coverage NGS strategies, validated bioinformatics approach, accurate variant assessment strategy, and confirmatory sequencing are prerequisites for reliable evaluation of *TTN* in clinical settings, and ideally with the inclusion of mRNA and/or protein level assessment for a definite diagnosis.

## Introduction

Titin (*TTN*) is a giant muscle protein expressed in the cardiac and skeletal muscles. It spans half of the sarcomere from Z-line to M-line. Titin is known to play a key role in muscle assembly, force transmission at the Z-line and maintenance of resting tension in the I-band region. The clinically relevant A-band of *TTN* binds to the thick filament, where it may regulate filament length and assembly, and is thought to be critical for biomechanical sensing and signaling. The C-terminal M-band contains a strain-sensitive kinase, which may have a role in cardiac signal transduction [1]. Before the era of next generation sequencing (NGS) only a small number of *TTN* gene mutations have been found to associate with cardiomyopathies [1–8]. This has largely been due to the difficulty to sequence large *TTN* gene with its ~363 exons. Consequently, *TTN* mutation frequency and therefore clinical impact were unknown.

Recently, using a combination of NGS and dideoxy sequencing, Herman et al. [9] estimated *TTN* truncating variants (TTNtv)—nonsense, frameshift and essential splice site, to be responsible for approximately 25% of familial cases of idiopathic dilated cardiomyopathy (DCM) and 18% of sporadic cases in a large cohort of subjects. Similarly to Herman et al. [9], in our recent study on Finnish patients with DCM, TTNtv were responsible for 20.6% of cases with family history of DCM. Furthermore, in both studies and others the mutations were not randomly distributed along the *TTN* gene as the bulk of the mutations were located predominantly in the A-band region and affecting all transcripts of *TTN* [10–14]. Another recent study confirmed that *TTN* truncations are highly enriched in DCM patients when compared to healthy controls [10]. Furthermore, identifying a truncating variant in the A-band region was estimated to have 93% risk of being disease causing. They also determined that C-terminal truncations affecting all transcripts were more pathogenic and mediated their effects through dominant negative mechanisms rather than haploinsufficiency. Roberts et al. [10] also confirmed that TTNtv-positive DCM patients manifest more severe clinical phenotypes than TTNtv-negative DCM patients. Recently, TTNtv have also been identified in patients with clinical features of both left ventricular non-compaction cardiomyopathy (LVNC) and dilated cardiomyopathy [15].

Utilization of reference population databases has significantly improved our ability to interpret genetic findings. Before the publishing of Exome Aggregation Consortium (ExAC) database in 2014, we have been dependent on the different versions of the 1000 Genomes Project and the National Heart Lung Institutes Exon Sequencing Projects (ESP) [16, 17]. Since the inception of 1000 Genomes in 2010, various phases, versions and releases of the project have evolved, with the phase 3 variants (which represents the final phase of the project) being based on data from 2535 individuals from 26 different populations around the world, with 60–100 individuals representing each population in the cohort. The quality and coverage of sequencing data in this database have varied significantly during the evolution of the database causing high prevalence of false positive insertion-deletions (INDELs) in earlier datasets. Recent effort by Golbus et al. [18] to estimate the prevalence of TTNtv in the population used the February 2012 release (phase 1 version 2) containing variants and phased genotypes across 1092 individuals from 14 different populations. In this study, the prevalence of frameshift INDELs that disrupt *TTN* was estimated to be as high as 3.2% (35/1092) among reference individuals. In the study by Roberts et al. [10], the prevalence of TTNtv was estimated 2% using a combination of 1000 Genomes call set (phase 1 version 3) and ESP call set.

Since the prevalence of TTNtv in reference populations is a critical determinant when interpreting genetic test results, we pursued to analyze this, for the first time, in the over 60 000 ExAC cohort together with the final version of 1000 Genomes project and ESP datasets. Our goal was also to define the specific prevalence of different TTNtv in various *TTN* domains to better understand the distribution and prevalence of different genetic findings. Our study reveals that identifying a frameshift, nonsense or an essential splice-site variant in the critical A-band of *TTN* is a rare event. In our study we conclude that in reference population only 1 in 1750 individuals carry a frameshift, 1 in 1100 carry a nonsense, and 1 in 2100 carry an essential splice variant in *TTN* A-band region that is estimated to affect all isoforms of the gene. We also determine the characteristics of nonsense variants identified and variability in the splice-site regions of *TTN* gene. We believe our results have a wide application in clinical interpretation of genetic test results and further emphasize that TTNtv in general population exceed significantly the prevalence of DCM associated with *TTN* truncations.

## Methods

### 1000 Genomes Project Data

*TTN* gene variant data from the various phases and versions of 1000 Genomes Project were retrieved, and truncating variants comprising of splice, nonsense and frameshift variants were used for analysis.

### Analysis of *TTN* Truncations in the Population

Considering the INDELs filtering on 1000 Genomes Project phase 1 variant call sets (ftp://ftp.1000genomes.ebi.ac.uk/vol1/ftp/release/20110521/README.phase1_integrated_release_version3_20120430) and the release of the phase 3 call set, we were keen to re-estimate the frequency of *TTN* truncating mutations in the population. We downloaded the exonic boundaries of *TTN* from the Ensembl Genome Browser (www.ensembl.org), and extracted exonic variants from the three versions of the phase 1 variants call sets (V1, V2, V3), and the version 5 (V5) of the phase 3 variants call set available in the 1000 Genomes Project database. Only calls that passed all quality filters were used for downstream analysis.

### Exome Sequencing Project (ESP) and Exome Aggregation Consortium (ExAC) Data

In a bid to capture a complete map of population *TTN* truncations and to further filter likely false positives in the latest release of 1000 Genomes Project, we utilized variants reported in two other well-annotated databases–Exome Sequecing Project (ESP) [17] and ExAC [19], with over 6500 and 60700 individuals respectively. As 1000 Genomes Project cohorts and ESP cohorts were part of the ExAC project cohorts, we filtered *TTN* truncating variants unique to either 1000 Genomes Project or ESP call sets. Variants located in novex-specific exons and exons specific to few transcripts of *TTN* were filtered before filtering for sequencing coverage/depth. See below for more details on the workflow and results.

## Results

### *TTN* Truncating Variants in the Evolution of 1000 Genomes Project

**Fig 1:**
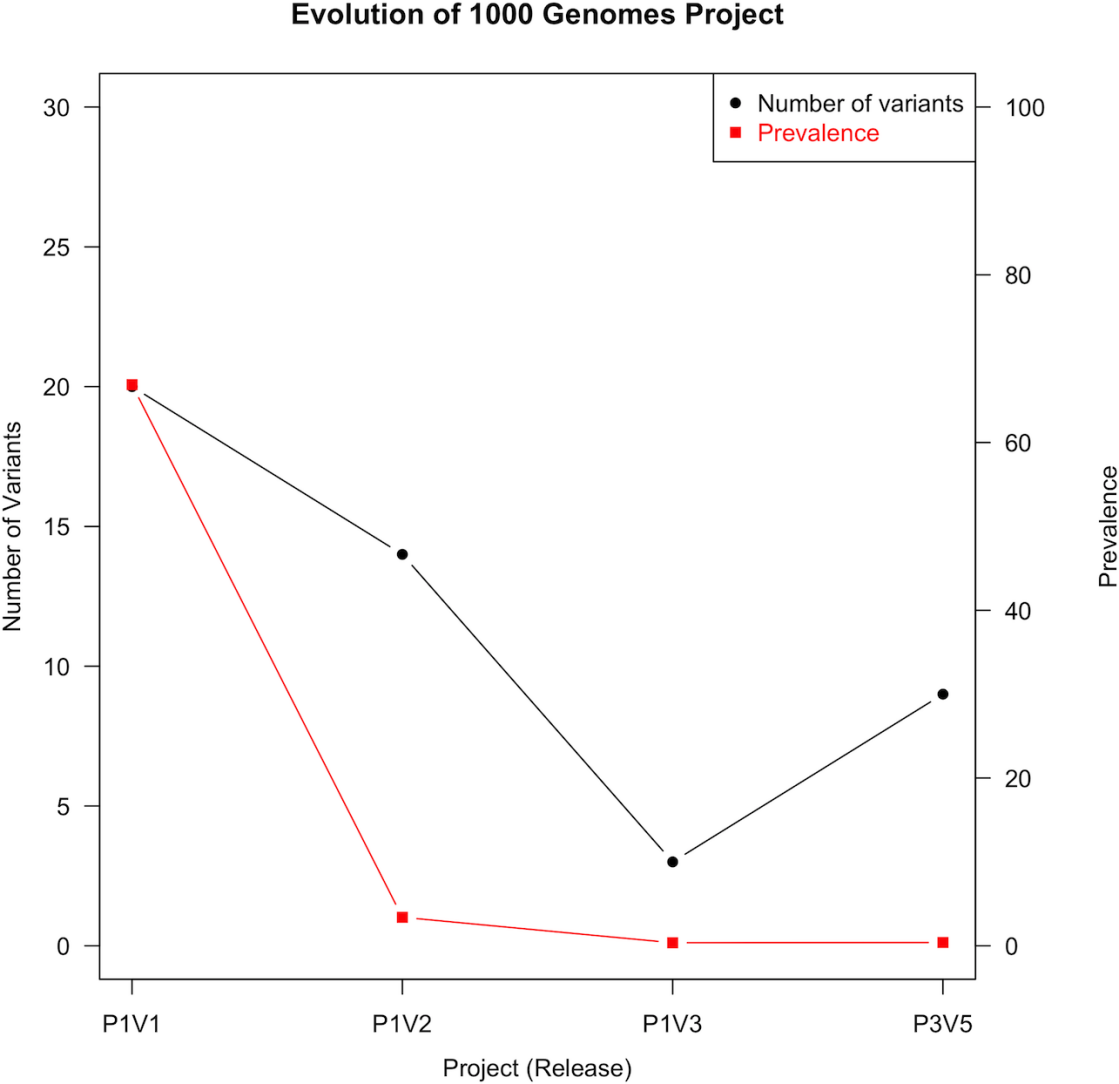
Influence of 1000 genomes project evolution on titin truncation prevalence. Abbreviations: P1V1 –phase 1 version 1; P1V2 –phase 1 version 2; P1V3 –phase 1 version 3; P3V5 –phase 3 version 5.

We extracted TTNtv that passed all quality filters as reported by the 1000 Genomes Project from various phases and versions of the project. Based on the total number of TTNtv in each version of the phase 1, and the version 5 of the phase 3 releases, we estimated the prevalence of TTNtv in the population. Interestingly, we observed a downward trend in both the number of TTNtv and TTNtv prevalence from the oldest to the latest release of the project (Fig 1). Version 2 of the phase 1 release contained predominantly rare TTNtv with high call qualities as against version 1 TTNtv composed of predominantly common TTNtv with very low qualities.

To demonstrate how the quality of sequencing and bioinformatic analysis contributes to our understanding of variant frequencies in general population, especially when estimating INDELS, we evaluated the detection rates of TTNtv in the evolution of 1000 Genomes project (see discussion) (Fig 1). The initial phase 1 (V1) integrated data contained 22 truncating mutations: 4 nonsense, 1 splice region, and 17 frameshift mutations (16 of which were single nucleotide (SN) INDELs) (S1 Table). After the first INDELs filtering on version 1 of the phase 1 integrated variant call set, six of the SN INDELs were filtered as false positives in the set due to technical artifacts introduced in the sequencing step. Version 2 phase 1 integrated variant call set contained 16 TTNtv: 4 nonsense, 1 splice region, and 11 frameshifts (10 SN INDELs) reported in Golbus et al. [18] study. All frameshift mutations as well as splice region variant were filtered out as false positives in the version 3 of the phase 1 integrated variant call set (S2 and S3 Tables). We identified thirty-three TTNtv in the phase 3 integrated variant call set of the 1000 Genomes Project: 1 frameshift, 13 splice and 19 nonsense. Interestingly, a vast majority of the splice site and nonsense variants were located in either novex-3 specific exon, exons either not expressed or with low expression in human left ventricle (LV), described by Roberts et al. [10] as proportion spliced-in (PSI) values or in exons not affecting all transcripts of the gene. Furthermore, several of the nonsense variants were located in regions with low coverage, suggesting they are likely false positives. In this analysis we demonstrate a marked reduction in the prevalence of TTNtv during the evolution and improvement of 1000 Genomes Project (Fig 1). The variants of the final phase (phase 3) were used in the analyses as our 1000 Genomes dataset.

### *TTN* Truncating Variants in General Population

**Fig 2:**
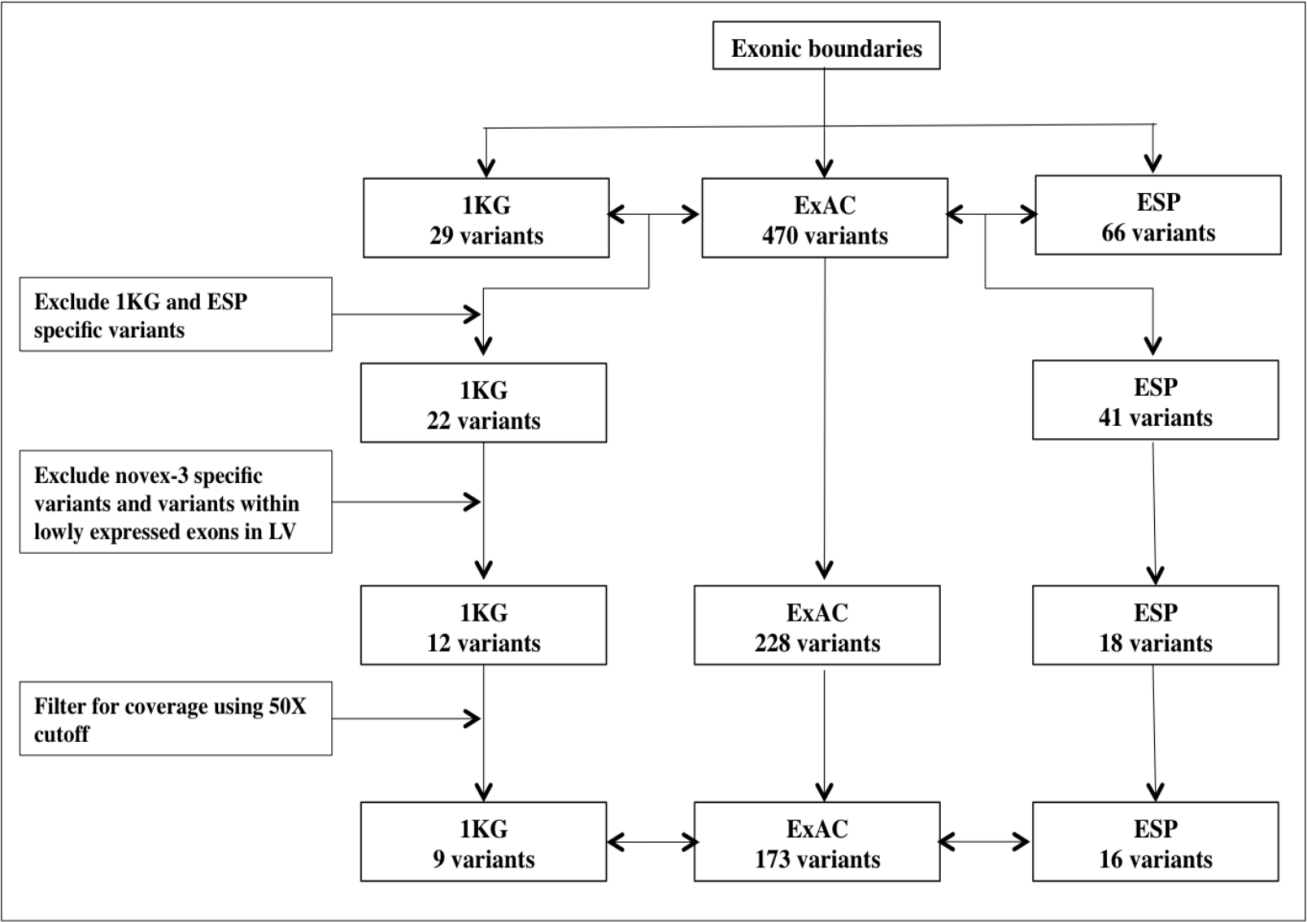
Flowchart for analysis of *TTN* truncations in publicly available reference populations. Abbreviations: 1KG– 1000 Genomes project; ESP–Exome Sequencing Project; ExAC—Exome Aggregation Consortium; LV–Left ventricle.

In our goal to accurately estimate the prevalence of TTNtv in the general population, we queried TTNtv in 1000 Genomes, ESP and ExAC databases (Table 1). The workflow is described in Fig 2. With our strategy, we were able to filter likely false positives unique to either 1000 Genomes or ESP variants. Of note, only three splice-site variants were shared between 1000 Genomes and ESP *TTN* truncation call sets suggesting that ESP is enriched with population specific variants, as it is made up of primarily Americans either of European (EA) or African (AA) origin. Furthermore, at least 52% of TTNtv (1000Genomes: 52% - 17/33; ESP: 54% - 36/66; ExAC: 53% - 247/470) found in the populations were located in exons that are not present in all transcripts and estimated to have low probability of pathogenicity.

**Table 1:** Distribution and burden of *TTN* truncations in publicly available reference populations.

	Total TTN truncating Allele			Excluding novex-3 specific, low PSI and likely false positive variants		
	1KG	ESP	ExAC	1KG	ESP	ExAC
Population size	2504	6504	60706	2504	6504	60706
Total TTNtv variant	29	66	470	9	16	173
Individuals	46	612	813	10	19	219
Prevalence (%)						
Population	1.837	9.409	1.339	0.399	0.292	0.360
**Mutation type # (Prevalence %)**						
Frameshift	1 (0.120)	25 (2.550)	179 (0.420)	1 (0.079)	3 (0.090)	63 (0.110)
Nonsense	15 (1.120)	28 (0.510)	177 (0.420)	7 (0.280)	11 (0.170)	72 (0.160)
Splice site	13 (0.600)	13 (6.350)	114 (0.490)	1 (0.040)	2 (0.030)	38 (0.090)
**Distribution by Domain # (Prevalence %)**						
Z-band	1 (0.039)	2 (0.030)	28 (0.062)	-	1 (0.015)	19 (0.037)
I-band	13 (0.718)	49 (8.225)	289 (0.957)	1 (0.039)	7 (0.107)	39 (0.072)
A-band	12 (0.918)	9 (0.153)	122 (0.252)	7 (0.319)	4 (0.061)	90 (0.196)
M-band	3 (0.119)	6 (0.999)	31 (0.067)	1 (0.079)	4 (0.107)	25 (0.054)
**Z-disk # (prevalence %)**						
Frameshift	-	-	10 (0.016)	-	-	8 (0.013)
Nonsense	-	1 (0.015)	8 (0.013)	-	1 (0.015)	4 (0.006)
Splice site	1 (0.039)	1 (0.015)	10 (0.032)	-	-	7 (0.018)
**I-band # (prevalence %)**						
Frameshift	-	17 (0.261)	109 (0.303)	-	-	10 (0.016)
Nonsense	4 (0.276)	20 (0.307)	103 (0.265)	1 (0.039)	5 (0.076)	19 (0.034)
Splice site	9 (0.434)	12 (0.184)	77 (0.388)	-	2 (0.030)	10 (0.021)
**A-band # (prevalence %)**						
Frameshift	1 (0.079)	4 (0.061)	50 (0.082)	1 (0.079)	1 (0.015)	35 (0.057)
Nonsense	10 (0.789)	5 (0.076)	49 (0.112)	6(0.239)	3 (0.046)	37 (0.090)
Splice site	1 (0.039)	-	23 (0.057)	-	-	18 (0.047)
**M-band # (prevalence %)**						
Frameshift	-	4 (0.061)	10 (0.023)	-	2 (0.030)	10 (0.006)
Nonsense	1 (0.039)	2 (0.030)	17 (0.034)	-	2 (0.030)	12 (0.024)
Splice site	2 (0.079)	-	4 (0.009)	1 (0.039)	-	3 (0.006)

Titin truncations in 1000 Genomes project (Phase 3), Exome Sequencing Project, and ExAC databases. The number of alleles and population prevalence for each variant is shown. Variants were filtered to exclude variants with low probability of pathogenicity (location in novex-specific and other exons with low expressions in LV). Abbreviations: 1KG– 1000 Genomes project; ESP–Exome Sequencing Project; ExAC—Exome Aggregation Consortium; PSI–Proportion Spliced-In

**Fig 3:**
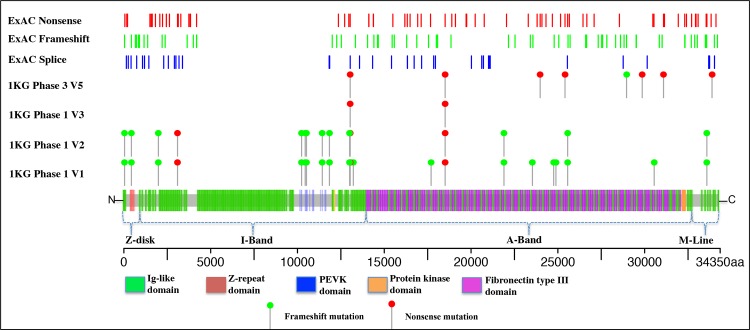
Spatial distribution of titin frameshift, nonsense and splice-site mutations in reference populations. Titin is linearly depicted with its 152 Ig-like domains in green and 132 fibronectin type III domains in purple. TTNtv are shown as lollipops and bars. Depicted are nonsense (red) and frameshift (green) mutations in various phases of the 1000 Genomes project. Dark grey bars represent the complete map of various *TTN* mutations from ExAC. Variants are shown relative to the titin Uniprot Sequence identifier Q8WZ42. The dashed lines below the protein schematic indicate the location of variants within the sarcomere. Abbreviations: 1KG– 1000 Genomes project; ESP–Exome Sequencing project; ExAC—Exome Aggregation Consortium; V–version.

When comparing the prevalence of TTNtv that affect all transcripts (excluding Novex-3 specific and low PSI exon variants) in ExAC vs 1000 Genomes, we identified lower frequency of nonsense mutations in ExAC cohort compared to 1000 Genomes cohort (0.16% vs. 0.28%, respectively) (Table 1). This was also the trend in nonsense variants identified only in the A-band region (0.09% vs. 0.23%) (Table 1). Essential splice site variants were very rare in ExAC database (0.047%) and were absent in 1000 Genomes and ESP. When evaluating the TTNtv that affect all transcripts in ExAC reference population by *TTN* band, 11.0% (19/173) are located in the Z-band; 22.6% (39/173) in the I-band; 52.0% (90/173) in the A-band; and 14.5% (25/173) in the M-band (Table 1 and Fig 3). Based on cohort size, the ExAC database gave the most reliable result in this analysis as some subtypes of TTNtv were absent in either 1000 Genomes or ESP call sets (Table 1). In the ExAC database the total prevalence of TTNtv affecting all transcripts was 0.36% (0.315% - 0.413%, 95% CI). Given that low coverage regions are often enriched with false-positive INDELs, we rendered the histogram of the TTNtv coverage data from ExAC and found out that about 80% of the variants have coverage of at least 50x. Moreover, we estimated TTNtv prevalence without introducing coverage filter and found out that despite the little increase in the number of variants, the prevalence did not increase significantly (0.46%) and falls within the confidence interval of the prevalence estimate with coverage filter.

The prevalence of TTNtv in clinically relevant A-band was 0.19%, suggesting that 1 in 500 individual carries one of the subtypes of TTNtv. When evaluating by mutation subtype, we conclude that in reference population, only 1 in 1100 individuals carries a nonsense mutation; 1 in 1750 carries a frameshift, and 1 in 2100 carries an essential splice site variant in *TTN* A-band region that is estimated to affect all transcripts.

### Distribution of *TTN* Truncating Variants by Ethnicity

Frequencies of TTNtv vary significantly between different ethnic groups (African population [AFR]: 0.46%, American population [AMR]: 0.50%, European population [EUR]: 0.27%, Asian population [AS]: 0.53%; *P* = 2.7 × 10^−5^). AS has the highest frequency of TTNtv while the European population has the least. The frequency of TTNtv in East Asian population (EAS), though lower, is not significantly different from that of the South Asian Population (SAS) (EAS: 0.37% vs. SAS: 0.62%; *P* = 0.091). Similarly, the frequency of TTNtv in Finnish European population (FIN) is lower but statistically indistinct when compared with the non-Finnish Europeans (NFE) (FIN: 0.12% vs. NFE: 0.28%%; *P* = 0.125).

Golbus et al. [18] recently reported that Asians have significantly more *TTN* protein altering variants (PAV) than all other ethnic groups (28.76 variants/individual). In this study, we have found a higher frequency of TTNtv in the Asians. Put together, this data confirms the enrichment of not only *TTN* PAV but also TTNtv in the Asian population (SAS in particular) compared to other ethnic groups.

### Nonsense *TTN* SNPs in Reference Population

We examined the patterns and the positions of the *TTN* nonsense SNPs in the population. Out of the twenty-three possible ways to change codons into stop codons (nine, seven and seven for the first, second and third positions, respectively), twenty-one were found in reference population, with tCg/tAg and tgT/tgA being absent. Nonsense SNPs were more frequent at the first codon position than at the second and third positions (*P* = 5.2 × 10^−11^, chi-square test). The most frequent type of nonsense mutation in the *TTN* gene was the change from Cga to Tga (R/*). Of note, the Cga/Tga transitional change at CpG mutation hotspots accounts for 91.3% (21/23) and 62% (21/34) of arginine residue nonsense mutations (R/*) at *TTN* A-band region and all *TTN* CpG hotspot nonsense mutations in the population data respectively. Interestingly, 36.1% (13/36) of R/* found in reference population have been reported in Catalogue of Somatic Mutations in Cancer (COSMIC) database [20].

### Splice Site Variants in Reference Population

**Fig 4:**
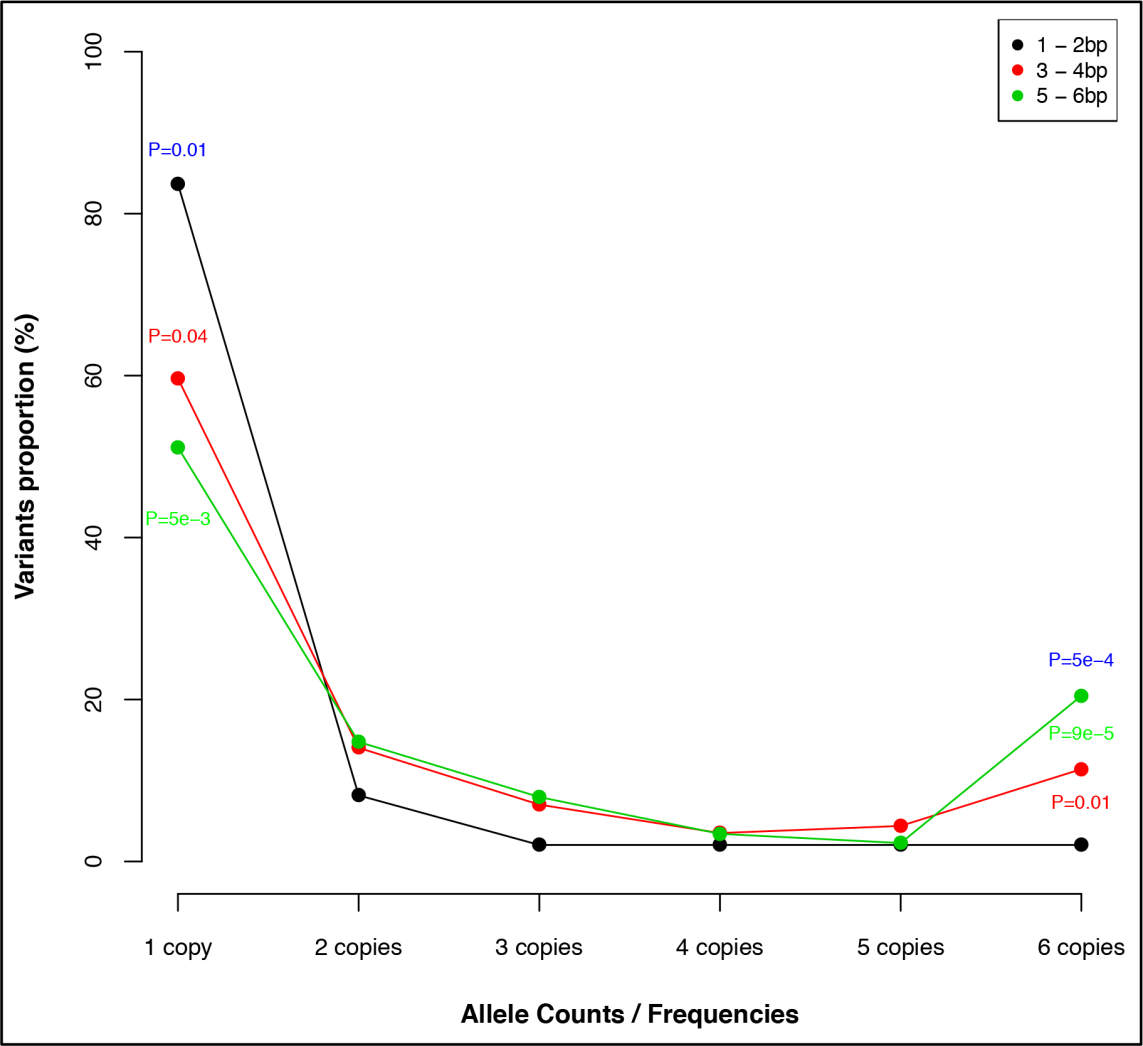
Titin population splice site/region allele frequency spectrum. *TTN* population non-essential splice-site variants (>2bp) have significantly lower proportion of private variants and higher proportion of low-frequency variants compared to essential splice-site variants (*P* = 0.01; *P* = 5.1 × 10^−4^, respectively). The P-values are shown for comparison between essential splice-site (1-2bp) and non-essential splice-site variants: 3–4bp (red), 5–6bp (green), and (blue) for combined comparison.

Of the total splice site/region variants, 86.9% (324/373) were non-essential splice variants located >2bp into the intronic region, with 52.8% (197/373) of the variants affecting *TTN* A-band exons. Of note, majority, 88.8% (175/197), of the splice variants affecting A-band exons were non-essential splice-site variants. When analyzing all essential splice site variants, we identified that 83.7% (41/49) were private. In contrast, non-essential splice-site variants had significantly lower proportion of private variants and higher proportion of low-frequency variants when compared to essential splice-site variants (*P* = 0.01; *P* = 5.1 × 10^−4^, respectively). The significance increases with increasing distance away from essential splice site (Fig 4).

## Discussion

By evaluating the ExAC reference population of over 60 000 individuals, we showed that TTNtv affecting all transcripts occur in 0.36% (0.315% - 0.413%, 95% CI) of the general population. These types of truncations in clinically relevant A-band can be detected in 0.19% of reference population. When evaluating the mutation types separately, the prevalence of each mutation type in A-band was lower than 0.1%, below a commonly used filtering criterion when searching for disease-causing mutation of rare dominantly inherited diseases. Furthermore, we provide insights on the position, type and frequency of nucleotide change(s) leading to nonsense mutations in *TTN*, and showed that non-essential splice-site variants in *TTN* have a tendency of being frequent in general population. In addition, we describe how increased quality of data analysis and larger number of reference individuals in 1000 Genomes Project has markedly reduced the prevalence of TTNtv in this database.

The 1000 Genomes Project published the pilot phase of the project in 2010. This release represents data from low-coverage (~2-fold– 6-fold) whole-genome sequencing of 179 individuals, high-coverage (~42-fold) whole-genome sequencing of 6 individuals in 2 trios, and exon-targeted sequencing (> 50-fold coverage) of 8140 exons in 697 individuals [16]. This was followed by Phase 1 project release that represents low coverage exome data available for the first 1092 samples. Phase 2 represents an expanded set of samples, around 1700 in number, and it was used for method development to both improve existing methods from phase 1. Furthermore, it was used to develop new methods to handle features like multi-allelic variant sites and true integration of complex variation and structural variants. The phase 1 integrated release, based on low coverage and exome data, contains phased genotypes for 1092 individuals from 14 populations, and the variants have been extensively used in several studies to draw conclusions on human genetic variations [18, 21, 22]. Although significantly improved, the data still contained higher fraction of frameshift INDELs in low-coverage regions suggesting high prevalence of false positive frameshifts. Subsequent filtering of these false positives resulted in version 2 (V2) and version 3 (V3) of the phase 1 integrated release. The false positive INDELs present in the February 2012 release used in Golbus et al. [18] study has led to high estimates of truncating *TTN* variants in reference population.

Nonsense mutations account for an appreciable proportion of single-basepair substitutions affecting gene-coding region reported in Human Gene Mutation Database (HGMD). Our analysis shows that as in other genes, Cga/Tga (Arg/*; resulting from methylation-mediated deamination) nucleotide substitution at CpG hotspot is the most frequent nucleotide substitution leading to nonsense mutation in *TTN*. The cytosine-guanine (CpG) dinucleotide, though under-represented in vertebrate genomes, has been reported to be a hotspot for pathological mutations in the human genome [23, 24]. This hypermutability is related to its role as the major site of cytosine methylation with the attendant risk of spontaneous deamination of 5-methylcytosine (5mC) to yield thymine [25]. In this study, we found a significant over-representation of Arg/* CpG hotspot mutation at *TTN* A-band region. Interestingly, 62% of Arg/* CpG hotspot mutations are located at *TTN* A-band. Furthermore, a significant proportion of *TTN* Arg/* CpG hotspot mutations have been reported as germ line mutations identified in various cancer cells. This highlights the fact that the CpG is a critical hotspot for both germline and somatic mutations. Considering the high prevalence of Arg/* CpG hotspots in A-band, majority of exons coding for A-band present in all *TTN* transcripts, and that nonsense mutations in this region do not lead to mRNA decay but seem to work through dominant negative mechanism, it is not surprising that the A-band region is vulnerable to truncating mutations that are highly enriched among DCM patients.

Epigenetic mechanisms could underlie the elevated mutation frequency in the *TTN* A-band region occurring also in the germ line and manifesting hereditary cardiomyopathy. Epigenetic mechanisms are increasingly being recognized as causes and modulators of human disease. To date, there are few studies on the contribution of DNA methylation to disease onset and progression in cardiomyopathy [26–28]. In 2011, Movassagh et al. [26] reported differential DNA methylation patterns in CG dinucleotides located at the promoter, intragenic and gene bodies CpG islands in human end-stage cardiomyopathy. Furthermore, using a single gene model, Meurs & Kuan [28] evaluated the methylation of the CpGs within the exon regions of the skeletal muscle isoform of the myosin binding protein C gene (*MYBPC2*) and cardiac myosin binding protein C gene (*MYBPC3*), a common causal gene for hypertrophic cardiomyopathy. Interestingly, the mean methylation level of CpGs was significantly higher in *MYBPC3* than *MYBPC2* (*P* < 0.0001). These probably suggest that there are unique aspects of this cardiac gene that may result in increased genetic mutability. As mechanisms promoting mutability play a role in disease prevalence, evaluation of the methylation levels of *TTN*, and other DCM-associated genes are warranted.

Differential splicing in regions with variable sarcomere ultrastructures accounts for the variable structures of *TTN* I-band in different tissues. In our goal to characterize essential splice and other splice region variants that might contribute to disease development, we examined and compared the position (1 - 6bp into the intronic region, 1 - 2bp being essential splice site variants) and frequency of all *TTN* essential splice-site and splice-region variants in the population. While essential splice-site variants were predominantly private, we observed an increase in allele copies of non-essential splice-site variants as the distance increases from the essential splice-site. Robert et al. [10] recently reported lower enrichment of noncanonical splice variants in DCM compared to canonical splice variants. Our results confirm that clinical interpretation of non-essential splice site variants in *TTN* gene is difficult and these variants should not be claimed deleterious without RNA level evidence of splicing defect.

Several studies have reported M-line *TTN* mutations in various skeletal muscle diseases including centronuclear myopathy (CM, MIM #160150) [29], tibial muscular dystrophy (TMD, MIM #600334) [30, 31], early-onset myopathy (MIM #611705) [6], distal myopathy and limb girdle muscular dystrophy 2J (LGMD2J, MIM #608807) [32], with or without cardiac muscle involvement. In 2007, Carmignac et al. [6] reported the first M-line homozygous titin truncations causing congenital titinopathy involving both cardiac and skeletal muscle. While most heterozygous M-line titin mutations cause late-onset, dominant disorders involving predominantly skeletal muscle, homozygous or compound heterozygous M-line *TTN* mutations cause early-onset, recessive muscle and cardiac disorder. M-line *TTN* mutations truncate only part of the M-band portion of *TTN* [6]. When such carboxy-terminal truncated titin proteins are integrated into the sarcomere, they cause recessive, early-onset skeletal and cardiac myopathy. In the case of DCM however, truncated titin proteins, though incorporated into the sarcomere, would not include the M-band residues, as TTNtvs causing DCM were clustered in *TTN* A-band but absent from Z-disk and M-band regions of *TTN*. The position of TTNtv causing DCM suggests a dominant negative effect. In addition, Herman et al. [9] argued that we would expect a more uniform distribution of such mutations if more proximal TTNtv caused DCM through haploinsufficiency. Furthermore, using RNA sequencing, Roberts et al. [10] reported a comparable total *TTN* transcript levels in patients with or without TTNtv. They also found and reported a comparable allelic expression of TTNtv and SNPs, which does not support substantial nonsense-mediated decay. Using a combination of genetic data, *TTN* RNA and protein expression in LV tissues, Roberts et al. [10] also concluded that TTNtv cause DCM by a dominant negative effect.

As majority of TTNtv have been identified at the heterozygous state in patients with sporadic or dominant conditions, such mutations are typically considered autosomal dominant. In majority of the cases, dominant inheritance has been clearly established by co-segregation. To the best of our knowledge, only a few studies have suggested recessive inheritance with *TTN* mutations. In these studies, TTNtv co-occurred with a second hit but actually only eight out of 18 patients had two TTNtv, and in two of these cases, bi-allelic status was not confirmed by parental testing [29, 33, 34]. However, Ceyhan-Birsoy et al. [29] did not evaluate the parents’ cardiac phenotype; Chauveau et al. [33] found normal echocardiography and ECG in all parents but they were relatively young (aged 38–55 years) and thus a genotype-positive yet a phenotype-negative status cannot be excluded. In the Evilä et al. [34] study, the other patient with bi-allelic TTNtv had an affected father with skeletal myopathy but the patient mother’s cardiac phenotypes were not evaluated. Interestingly, all TTNtv reported in these studies were located in the M-line of *TTN*. These observations together with our prevalence estimates highlight that recessive cardiac disease caused by titin dysfunction is possible in rare occasions. Initial evidence exists for recessive inheritance of *TTN* mutations in neurological disease although clearly more comprehensive segregation studies are warranted to ease interpretation of TTNtv.

## Concluding Remarks

Considering the accumulated evidence of *TTN* truncating mutations being the most common cause for hereditary DCM [9–11] together with our observations on low prevalence of potentially disease-causing TTNtv in general population, we emphasize that identifying a TTNtv, especially in the A-band region and affecting all transcripts, have a potentially higher risk of being disease causing than previously anticipated [10]. Further studies are required to elucidate the full phenotypic spectrum of TTNtv-associated myocardial disease. It is likely that the severe end-stage DCM or LVNC only represent the peak of the iceberg and milder disease forms that will never develop fulminant cardiomyopathy may exist e.g. transient peripartum cardiomyopathy. This information is needed as it affects our interpretation of genetic variants in reference populations and also the genetic counseling processes and clinical management. Evaluating the *TTN* gene reliably in clinical setting requires expertise in DNA library preparation, high coverage NGS strategies, validated bioinformatics tools, confirmatory sequencing, skilled interpretation team, and ideally the inclusion of mRNA and/or protein level assessment for definite diagnosis. Current low coverage exome and whole genome sequencing approaches may be sub-optimal for diagnostic work-up in cardiomyopathies as their efficacy for identifying INDELs is weak compared to high coverage targeted sequencing strategies [35, 36]. Moreover, the size of *TTN* gene makes it more susceptible than an average sized gene to false positive truncating variant especially when using ‘clinical’ exome sequencing. It can be easily demonstrated by the fact the there is four homozygous *TTN* variants (positions: 2:179610726, 2:179612587, 2:179571683, 2:179466515) in ExAC database and they are found as heterozygote in 1, 5, 10 and 10 individuals respectively, although we would expect to find 245 heterozygotes for one homozygote in this cohort. This indicates a high probability of detected homozygotes being false positives. Further studies are needed to evaluate the reliability of our reference data, and especially to find out how many of the TTNtv in reference populations are true positives. It should be kept in mind that one out of seven low quality score variants (QS<500) in exome sequencing are likely false positives (non-detectable by Sanger sequencing) [37].

## Supporting Information

S1 Table Truncating *TTN* mutations identified in 1000 Genomes Project Cohort (P1V1). Marked (†) are the false positives that were filtered out in the subsequent version. Abbreviations: P1V1 –phase 1 version 1; GMAF–Global minor allele frequency.
(DOCX)

S2 Table Truncating *TTN* mutations identified in 1000 Genomes Project Cohort (P1V2). Marked (†) are the false positives that were filtered out in the subsequent version. Abbreviations: P1V2 –phase 1 version 2; GMAF–Global minor allele frequency.
(DOCX)

S3 Table Truncating *TTN* mutations identified in 1000 Genomes Project Cohort (P1V3). Marked (†) are the variants that were not detected in the phase 3 integrated variant data. Abbreviations: P1V3 –phase 1 version 3; GMAF–Global minor allele frequency.
(DOCX)
