# Supplementary material for: Prevalence of Titin Truncating Variants in General Population
Source: PLoS One. 2015 Dec 23;10(12):e0145284. doi: 10.1371/journal.pone.0145284 (PMC4689403; doi:10.1371/journal.pone.0145284)
Supplement: S2 Table — Marked (†) are the false positives that were filtered out in the subsequent version. Abbreviations: P1V2 –phase 1 version 2; GMAF–Global minor allele frequency. (DOCX) [file pone.0145284.s002.docx]

**S2 Table**. **Truncating *TTN* mutations identified in 1000 Genomes Project Cohort (P1V2).**

| Location | Transcript | Nucleotide Change | Amino Acid Change | GMAF (N=2184) |
| --- | --- | --- | --- | --- |
| 2:179395288 | ENST00000589042 | c.106054delT | p.Ser35352Leufs*53 | 0.0032 (7)† |
| 2:179428912-179428913 | ENST00000589042 | c.81946dupC | p.Leu27316Profs*2 | 0.0005 (1)† |
| 2:179439949 | ENST00000589042 | c.70910delG | p.Gly23637Alafs*29 | 0.0005 (1)† |
| 2:179455726 | ENST00000589042 | c.60726T>A | p.Tyr20242* | 0.00019 (1) |
| 2:179494977 | ENST00000589042 | c.44272C>T | p.Arg14758* | 0.00019 (1) |
| 2:179495075-179495076 | ENST00000589042 | c.44173_44174delGA | p.Glu14725Argfs*27 | 0.0014 (3)† |
| 2:179505311-179505312 | ENST00000589042 | c.40679dupC | p.Thr13561Tyrfs*11 | 0.0005 (1)† |
| 2:179517380-179517382 | ENST00000589042 | c.39041_39042delAA | p.Lys13014Serfs*11 | 0.0005 (1)† |
| 2:179532190 | ENST00000589042 | c.35692A>T | p.Arg11898* | 0.0009 (2) |
| 2:179548796 | ENST00000589042 | c.32736delG | p.Lys10913Argfs*10 | 0.0005 (1)† |
| 2:179549673-179549674 | ENST00000589042 | c.32513dupC | p.Ala10839Cysfs*5 | 0.0018 (4)† |
| 2:179554281 | ENST00000589042 | c.31888delA | p.Thr10630Profs*3 | 0.0005 (1)† |
| 2:179632509 | ENST00000589042 | c.9448C>T | p.Arg3150* | 0.0005 (1) |
| 2:179640461-179640462 | ENST00000589042 | c.6129dupA | p.Glu2044Argfs*4 | 0.0009 (2)† |
| 2:179658211-179658212 | ENST00000589042 | c.1455dupA | p.Ala486Serfs*26 | 0.0027 (6)† |
| 2:179666885-179666886 | ENST00000589042 | c.274dupA | p.Thr92Asnfs*3 | 0.0037 (8)† |

Marked (†) are the false positives that were filtered out in the subsequent version. Abbreviations: P1V2 – phase 1 version 2; GMAF – Global minor allele frequency.
