# Supplementary material for: Prevalence of Titin Truncating Variants in General Population
Source: PLoS One. 2015 Dec 23;10(12):e0145284. doi: 10.1371/journal.pone.0145284 (PMC4689403; doi:10.1371/journal.pone.0145284)
Supplement: S3 Table — Marked (†) are the variants that were not detected in the phase 3 integrated variant data. Abbreviations: P1V3 –phase 1 version 3; GMAF–Global minor allele frequency. (DOCX) [file pone.0145284.s003.docx]

**S3 Table**. **Truncating *TTN* mutations identified in 1000 Genomes Project Cohort (P1V3).**

| Location | Transcript | Nucleotide Change | Amino Acid Change | GMAF (N=2184) |
| --- | --- | --- | --- | --- |
| 2:179455726 | ENST00000589042 | c.60726T>A | p.Tyr20242* | 0.0005 (1) |
| 2:179494977 | ENST00000589042 | c.44272C>T | p.Arg14758* | 0.0005 (1) |
| 2:179532190 | ENST00000589042 | c.35692A>T | p.Arg11898* | 0.0009 (2)† |
| 2:179632509 | ENST00000589042 | c.9448C>T | p.Arg3150* | 0.0005 (1) |

Marked (†) are the variants that were not detected in the phase 3 integrated variant data. Abbreviations: P1V3 – phase 1 version 3; GMAF – Global minor allele frequency.
